# Molecular Basis for Bordetella pertussis Interference with Complement, Coagulation, Fibrinolytic, and Contact Activation Systems: the Cryo-EM Structure of the Vag8-C1 Inhibitor Complex

**DOI:** 10.1128/mBio.02823-20

**Published:** 2021-03-23

**Authors:** Arun Dhillon, Justin C. Deme, Emily Furlong, Dorina Roem, Ilse Jongerius, Steven Johnson, Susan M. Lea

**Affiliations:** a Sir William Dunn School of Pathology, Oxford, United Kingdom; b Central Oxford Structural Molecular Imaging Centre, Oxford, United Kingdom; c Sanquin Research, Department of Immunopathology, and Landsteiner Laboratory, Amsterdam University Medical Centre, Amsterdam Infection and Immunity Institute, Amsterdam, the Netherlands; d Department of Pediatric Immunology, Rheumatology, and Infectious Diseases, Emma Children’s Hospital, Amsterdam University Medical Centre, Amsterdam, the Netherlands; Columbia University

**Keywords:** *Bordetella pertussis*, immune evasion, complement system, serpin, autotransporters, C1-INH, bacterial pathogenicity, single-particle cryo-EM, three-dimensional structure

## Abstract

Complement, contact activation, coagulation, and fibrinolysis are serum protein cascades that need strict regulation to maintain human health. Serum glycoprotein, a C1 inhibitor (C1-INH), is a key regulator (inhibitor) of serine proteases of all the above-mentioned pathways. Recently, an autotransporter protein, virulence-associated gene 8 (Vag8), produced by the whooping cough pathogen, Bordetella pertussis, was shown to bind to C1-INH and interfere with its function. Here, we present the structure of the Vag8–C1-INH complex determined using cryo-electron microscopy at a 3.6-Å resolution. The structure shows a unique mechanism of C1-INH inhibition not employed by other pathogens, where Vag8 sequesters the reactive center loop of C1-INH, preventing its interaction with the target proteases.

## Abstract (precis)

The structure of a 10-kDa protein complex is one of the smallest to be determined using cryo-electron microscopy at high resolution. The structure reveals that C1-INH is sequestered in an inactivated state by burial of the reactive center loop in Vag8.

## INTRODUCTION

Protein cascades coordinate key processes for health within human serum, in particular immune and inflammatory responses (complement and contact activation) and control of clotting (contact activation, coagulation, and fibrinolysis) (1–3). Although they are independent processes, coordination between the pathways occurs by shared regulation, particularly by C1 inhibitor (C1-INH) (4). C1-INH inhibits serine proteases involved in activation and control of these systems by formation of protease–C1-INH complexes such that the level of these complexes is directly proportional to the level of *in vivo* activation of all four systems (5). C1-INH is established as a key regulator of complement via inhibition of the activation proteases C1r, C1s, mannan-binding lectin serine protease 1 (MASP-1), and MASP-2 and is the dominant inhibitor of plasma kallikrein (contact activation system), coagulation factors XIIa and XIa, and thrombin (6–10). The mode of inhibition of these proteases involves interaction between the reactive center loop (RCL) of the C-terminal serpin domain of C1-INH to form a covalently linked acyl-enzyme complex that distorts the enzyme active site and is irreversibly bound (11–13). Additionally, C1-INH has been implicated in regulation of fibrinolysis via action against tissue-type plasminogen activator (tPA) and plasmin, although study of this is complicated by the fact that both these enzymes also cleave C1-INH (14, 15), highlighting the view that the serpin mechanism is a balancing act between trapping the enzyme in a nonfunctional complex with the inhibitor and cleavage of the inhibitor by the target enzyme.

Whooping cough (pertussis) is an infectious disease of the respiratory system caused by the Gram-negative bacterium Bordetella pertussis (16). B. pertussis employs a range of virulence factors to colonize the human host and evade immune responses (17). Some of these factors, e.g., virulence-associated gene 8 (Vag8), *Bordetella* resistance to killing A (BrkA), filamentous hemagglutinin (FHA), and B. pertussis autotransporter protein C (BapC), have been implicated in evasion of the complement system (18–21). While the mechanisms of action of BrkA, BapC, and FHA are still unclear, Vag8, a 95-kDa autotransporter protein, was recently shown to interfere with the complement and contact systems by binding to C1-INH, leading to bacterial complement evasion (22, 23). Autotransporters represent the type V bacterial secretion system and possess a C-terminal membrane-embedded β-barrel domain that facilitates the translocation of the N-terminal passenger domain, responsible for effector functions, across the outer membrane (24). In the case of Vag8, the cleaved N-terminal domain has been detected in bacterial culture supernatant in addition to the full-length Vag8 being presented on outer membrane vesicles (OMVs) and on the cell surface (22). Deletion of the gene encoding Vag8 predisposes B. pertussis to complement-mediated killing (18, 22).

Although C1-INH is an inhibitor of complement activation, targeting C1-INH activity is used as a strategy for complement evasion by a range of different pathogens. Streptococcus pyogenes and Legionella pneumophila use enzymes, SpeB and ChiA, respectively, to cleave C1-INH (25, 26), while Plasmodium falciparum, Borrelia recurrentis, and Salmonella enterica serovar Typhimurium depend on *Pf*MSP3.1, CihC, and lipopolysaccharide (27–29), respectively, to capture C1-INH on the cell surface, maintaining it in an active, inhibitory state. A hybrid of the above two strategies of C1-INH targeting has been proposed to be used by Escherichia coli O157:H7, involving capture of C1-INH on the cell surface followed by an enzymatic cleavage (30). While targeting an inhibitor to the pathogen surface is a self-evident way of enhancing immune evasion, the utility of destruction of C1-INH is less obvious, but it is explained by the fact that removal of C1-INH from serum leads to rapid, catastrophic activation of complement, leading to depletion of complement activity and so, perversely, less complement attack on the pathogen (22, 25, 26).

Globally, pertussis is responsible for a large number of infant deaths, especially in low-income countries, and is a financial burden even in developed economies (31, 32). Despite extensive vaccination programs, B. pertussis infections are on the rise again (33). Reasons to explain the rising infections have been contentious and include waning of immunity generated by acellular pertussis vaccines and evolution of more pathogenic strains (34–37); therefore, a molecular understanding of the mode of action of B. pertussis virulence factors such as Vag8 is desirable. More broadly, with evidence mounting that activation of coagulation and excessive cytokine release are key drivers of COVID-19 pneumonia and mortality, with contact activation appearing to be particularly important in driving pathological upregulation of inflammatory mediators and coagulation, interest in pathogenic mechanisms acting on these systems is further increased (38–41).

To that end, we have determined the structure of the Vag8 passenger domain in complex with the C1-INH serpin domain using single-particle cryo-electron microscopy (cryo-EM) to a resolution of 3.6 Å. The cryo-EM structure of this complex reveals that Vag8 noncovalently sequesters the RCL of C1-INH in the groove of the elongated passenger domain, preventing C1-INH–protease interactions and regulation. Thus, B. pertussis overrides complement regulatory control by a unique mechanism not previously seen in other pathogens. Sequestration of C1-INH in this manner not only leads to complement evasion but also promotes kallikrein activation, leading to increased levels of the vasoactive peptide bradykinin, increased fibrinolysis, and coagulation. Thus, B. pertussis widely perturbs serum activities across a broad spectrum by production of a single protein molecule.

## RESULTS

**FIG 1:**
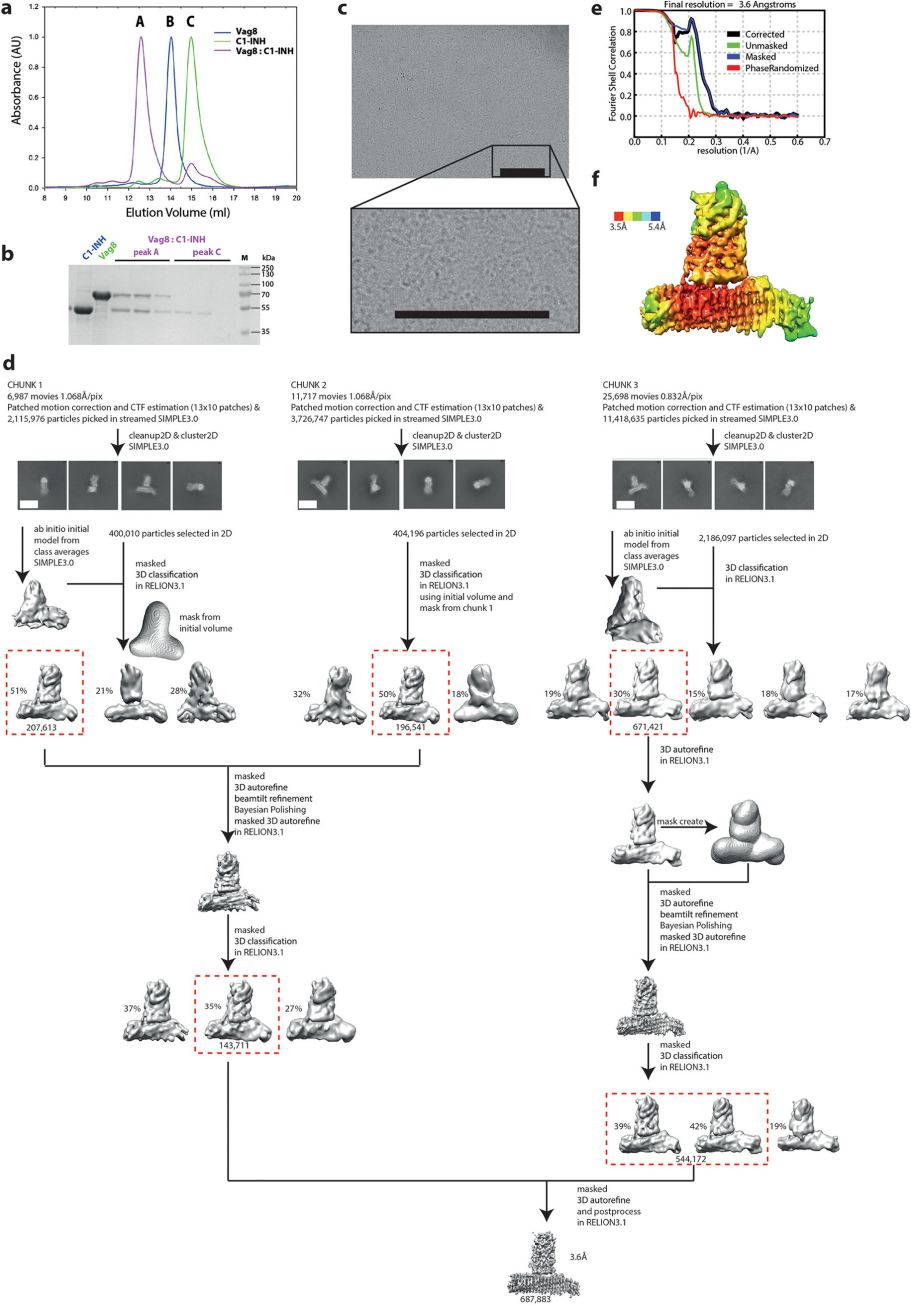
Determination of the single-particle cryo-EM structure of the Vag8–C1-INH complex at 3.6 Å (a) Size exclusion chromatography analysis shows that Vag8 binds C1-INH to form a complex (purple). One hundred microliters of an approximately 1:2 molar ratio of Vag8 to C1-INH was mixed and purified using an S200 Increase chromatography column. A, B, and C indicate the locations at which Vag8–C1-INH complex, Vag8, and C1-INH, respectively, elute. (b) Fractions under peaks A and C from Vag8–C1-INH purification when run on 15% (wt/vol) SDS-PAGE gel confirm that the peak at location A contains Vag8 bound to C1-INH, while unbound C1-INH elutes in peak C. (c) Representative micrograph of Vag8–C1-INH complex on a carbon-coated grid. Bar, 200 Å. (d) Cryo-EM data of Vag8–C1-INH complex were collected and initially processed as 3 different chunks (chunks 1, 2, and 3) and combined at later stages during processing using SIMPLE 3.0 and RELION 3.1. Masked 3D classification of chunk 2 data was done using the initial volume and mask from chunk 1. Subsequently, selected particles from chunk 1 and 2 were combined, and masked 3D classification was performed. Selected particles from this data set were combined with selected particles from chunk 3 data obtained after masked 3D autorefinement and masked 3D classification. This final combined data set was then autorefined and postprocessed in RELION 3.1, resulting in a 3.6-Å volume. Bar for 2D averages, 50 Å. (e) Gold-standard FSC curves of Vga8:C1-INH complex volumes postprocessed in RELION 3.1. Curves: red, phase randomized; green, unmasked; blue, masked; black, corrected. (f) Volume colored by estimated local resolution (Å).

To better understand how B. pertussis subverts C1-INH function, we heterologously expressed and purified both the passenger domain of Vag8 and the serpin domain of C1-INH (Fig. 1a and b). When mixed at an approximately equimolar ratio the proteins formed a complex that could be separated from a small amount of residual isolated C1-INH by size exclusion chromatography (Fig. 1a and b). This Vag8–C1-INH complex was then concentrated to 0.5 mg/ml and applied to Quantifoil R1.2/1.3 carbon-coated grids before blotting using a Mark IV Vitrobot and plunge freezing in liquid ethane. Single-particle cryo-EM data were collected using a Titan Krios at 300 kV equipped with a Gatan BioQuantum and K3 detector, as described in Materials and Methods. The small size of the complex (∼100 kDa) meant that individual particles were difficult to discern at the micrograph level (Fig. 1c); however, manual picking of ∼1,000 particles followed by 2D classification generated 2D averages that were used for automated picking of more than 40,000 movies, collected from three grids (Fig. 1d). Data were processed as shown in the workflow (Fig. 1d) using both SIMPLE 3.0 (42) and RELION 3.1 (43) to yield a final volume based on 687,883 particles with an estimated resolution (by gold-standard Fourier shell correlation (FSC), 0.143 criterion) of 3.6 Å (Fig. 1e). Calculation of a local resolution-filtered volume (Fig. 1f) (RELION 3.1) (43) demonstrates that the core of Vag8 and size of interaction with C1-INH are well defined, with a resolution estimate of 3.5 Å despite the small size of this complex placing it among the 10 smallest structures determined to date using this method (44).

**FIG 2:**
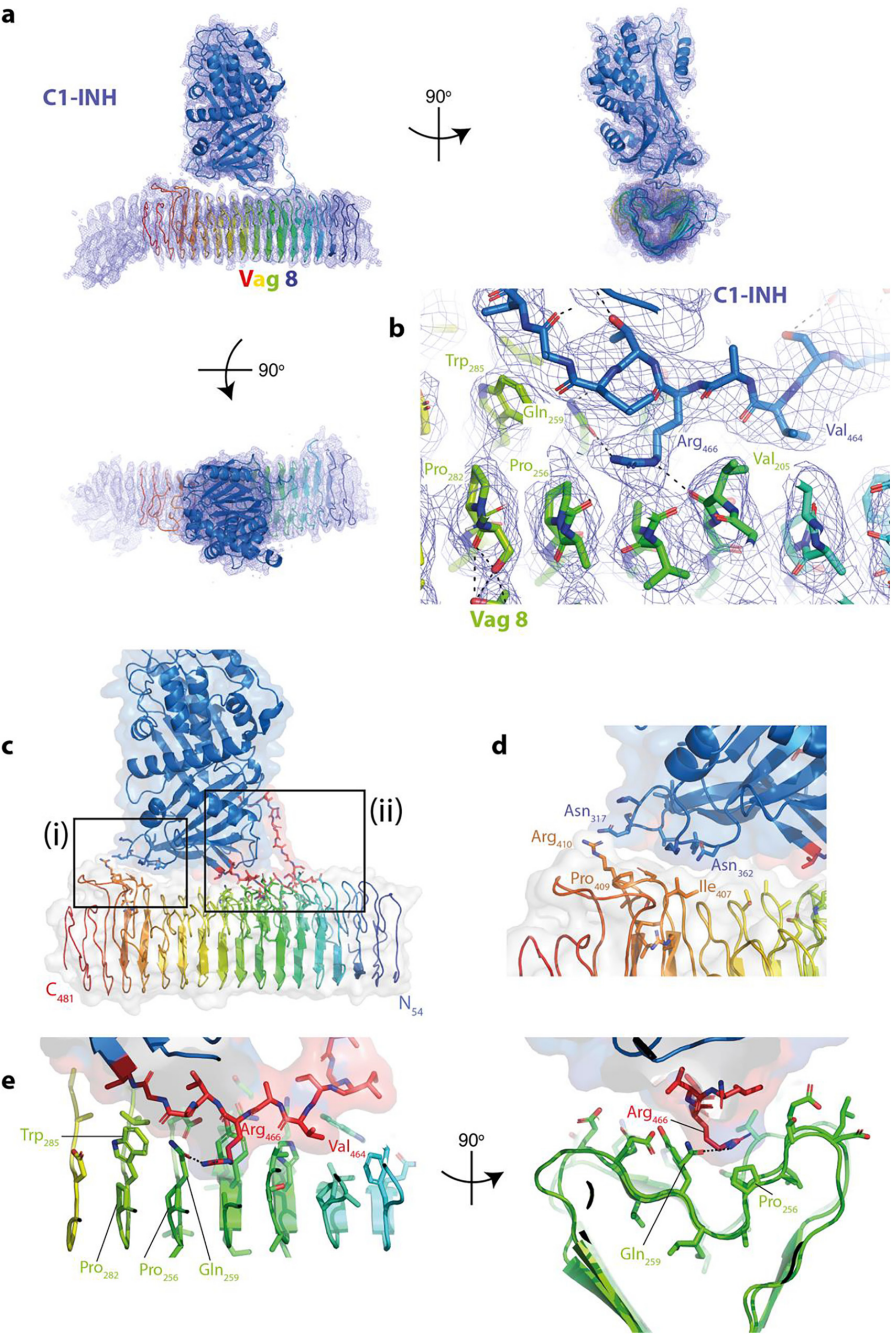
Structure of Vag8–C1-INH complex. (a) Views of the model of Vag8–C-INH in the experimental volume. Both proteins are shown in a cartoon representation, with Vag8 colored from blue at the N terminus to red at the C terminus and C1-INH colored blue. Volume is contoured at 3σ. The figure was drawn using PyMOL (The PyMOL Molecular Graphics System, version 2.0; Schrodinger, LLC). (b) Closeup of the central portion of the C-INH RCL in the Vag8 cleft, with key residue interactions highlighted. (c) Overview of the complex with the two points of contact boxed. (d) Closeup of the interactions in the smaller contact site (box i in panel c). (e) Two views from the top and end of the complex of the larger interaction site (box ii in panel c).

A *de novo* model was built manually using the program COOT (45) for the region from position 54 to 481 of Vag8. Although residual density could be seen in the volume both N- and C-terminal to this region (Fig. 2a), it was not possible to build an atomic model for residues 40 to 53 and 482 to 610. The model of the active form of the C1-INH serpin domain (46) was placed and remodeled to fit the volume, with the only major changes in conformation being within the RCL, which is seen to be sequestered within the cleft of the Vag8 beta-barrel fold. Figure 2b shows the quality of the volume around key side chains within the binding site. Further cycles of manual rebuilding and real-space refinement in PHENIX (47) led to the generation of the model presented in Fig. 2 and described in Table 1.

**TABLE 1:** Structure solution and model quality for human C1 inhibitor complex with B. pertussis Vag8 (EMDB 11814; PDB code 7AKV)

Parameter	Value^a^	
Data collection and processing		
Magnification	81,000	105,000
Voltage (kV)	300	300
Electron exposure (e^–^/Å^2^)	55.6	59.6
Defocus range (μm)	0.5–2.5	0.5–2.5
Pixel size (Å)	1.068 physical pixel (0.534 superresolution)	0.832 physical pixel (0.416 superresolution)
Symmetry imposed	C1	C1
Initial particle images (no.)	5,842,723	11,418,635
Final combined particle images on 0.832 Å pixel scale (no.)	687,883	
Map resolution (Å)	3.6	
FSC threshold	0.143	
Map resolution range (Å)	3.5–5.4	

Refinement		
Initial model used (PDB code)	C1-INH, 5DU3; Vag8, none	
Model resolution (Å)	3.6	
FSC threshold	0.143	
Model resolution range (Å)	3.5–5.4	
Map sharpening B factor (Å^2^)	−107	
Model composition		
Nonhydrogen atoms	6,203	
Protein residues	799	
Ligands	0	
B factors (Å^2^)		
Protein	49	
Ligand	NA	
RMS deviations		
Bond lengths (Å)	0.004	
Bond angles (°)	0.631	
Validation		
MolProbity score	3.05	
Clashscore	74.5	
Poor rotamers (%)	0.16	
Ramachandran plot		
Favored (%)	82.2	
Allowed (%)	17.8	
Disallowed (%)	0.0	
Map to model FSC		
0.5 criterion (Å)	3.8	
0.143 criterion (Å)	2.9	

a First column: data collection parameters for chunks 1 and 2. Second column: data collection parameters for chunk 3. Lower in the table, the single column is for refinement because the data were combined to yield one volume and hence one atomic model.

The model for the complex reveals that C1-INH associates with the cleft within the Vag8 passenger domain beta-barrel, with two contact sites (Fig. 2c). The first involves contacts between two loops at the base of the C1-INH serpin domain (around residues 317 and 362) and one of the longer loops incorporated in the Vag8 beta barrel (residues 407 to 410) (Fig. 2d). This is a fairly small contact area, burying approximately 100 Å^2^ on each protein. The other point of contact is a much more significant interaction which buries the side chains of the majority of the RCL residues between 461 and 474 within the Vag8 beta-barrel cleft, burying ∼600 Å^2^ on both components (Fig. 2e).

**FIG 3:**
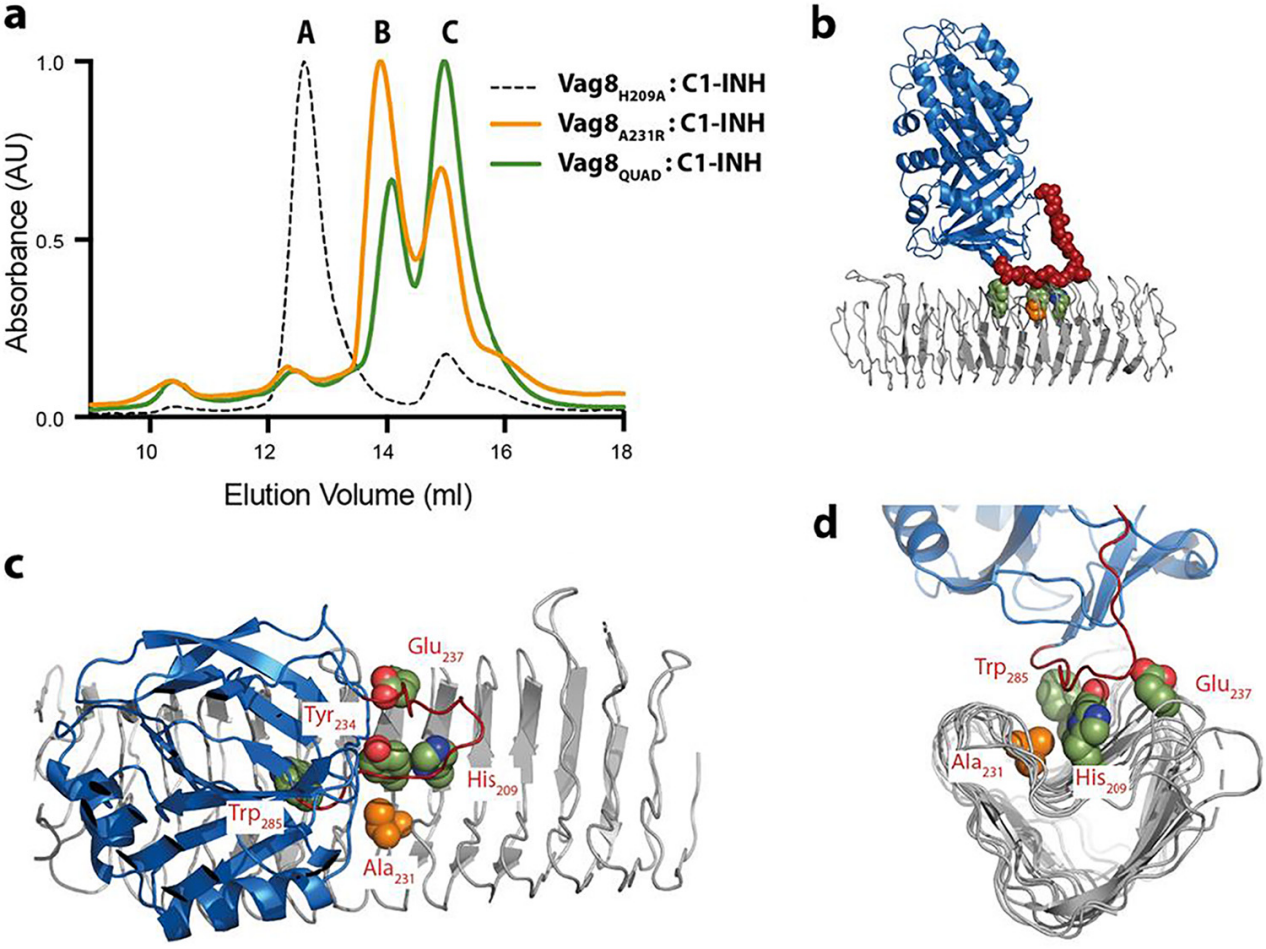
Mutation of residues within the interface abolishes complex formation. (a) One hundred microliters of an approximately 1:2 molar ratio of Vag8–C1-INH was mixed and purified using an S200 Increase chromatography column. A, B, and C indicate the locations at which Vag8–C1-INH complex, Vag8, and C1-INH, respectively, elute. Vag8_H209A_ (dashed line) contains one of the mutations that make up the Vag8_QUAD_ set and is still capable of forming a complex with C1-INH (as are the other mutations that form the Vag8_QUAD_ set in isolation; data not shown), whereas both Vag8_QUAD_ (green) and Vag8_A231R_ (orange) do not form any complex with C1-INH under these conditions, and the two mixed components elute independently in peaks B and C. (b to d) Views of the Vag8–C1-INH complex, with Vag8 shown as a gray cartoon, C1-INH as a blue cartoon, residues and mutated residues as space-filling spheres with carbons colored to reflect the color scheme in panel a. In panel b, the C1-INH RCL is colored dark red, and the main-chain atoms are shown as spheres. Panels b to d were drawn using PyMOL (The PyMOL Molecular Graphics System, version 2.0; Schrodinger, LLC).

To further probe the interactions seen in the complex, we designed single and multiple point mutations in Vag8 to test their effect on complex formation. Mutant forms of Vag8 were expressed, purified, and then mixed with the C1-INH serpin domain, and complex formation was assayed by size exclusion chromatography (Fig. 3a; Table 2). With the exception of a mutation designed to sterically block binding of the RCL in the cleft by replacement of a small alanine side chain with a very large arginine side chain (A231R) (Fig. 3; also data not shown), mutation of multiple residues within the cleft to alanine was required to prevent formation of the complex, emphasizing the extended nature of the interaction site (Fig. 2 and 3).

**TABLE 2:** Binding analysis of Vag8 mutants by size exclusion chromatography

Vag8 mutation(s)	Phenotype (C1-INH binding activity)
H209A	+
A231R	−
Y234A	+
E237A	+
W285A	+
H209A Y234A E237A W285A	−

a +, Vag8–C1-INH complex peak was seen; −, proteins eluted separately and no complex peak was observed.

## DISCUSSION

B. pertussis targets regulation of immune, inflammatory, and clotting processes by scavenging C1-INH using the passenger domain of Vag8. Our structure reveals that formation of this complex directly impacts the physiological systems by masking the RCL required for C1-INH to carry out its inhibitory activities within the bacterial protein. Unlike the native function of C1-INH, which results in formation of a covalent link between the RCL and the target, the inhibitor complex buries the RCL within the cleft of the bacterial protein via noncovalent interactions. Formation of a stable complex involving the RCL sterically occludes C1-INH interactions with its physiological partners.

For the complement system, the strategy of releasing inhibition, thus driving destruction of activity by overactivation, is surprisingly common, with a variety of organisms attacking regulators at all different levels within the cascade, e.g., by proteolytic inactivation (reviewed in reference 48).

In contrast to Plasmodium falciparum, Borrelia recurrentis, and *Salmonella* Typhimurium, which capture C1-INH on their surface in an active state (27–29), we see here how B. pertussis sequesters the protein away from its natural inhibitory role, regulating complement by overactivation. It remains to be seen if other C1-INH-binding organisms use a similar strategy.

## MATERIALS AND METHODS

### Expression and purification of Vag8.

Cloning of the Vag8 passenger domain (residues 40 to 610) into a modified pRSETb plasmid has been reported previously (22). The recombinant plasmid was transformed into Escherichia coli C41(DE3) cells, which were then plated on LB agar plates supplemented with 50 μg/ml ampicillin. Protein production was carried by growing E. coli C41(DE3) cells expressing Vag8pd in LB medium supplemented with 50 μg/ml ampicillin at 37°C and 180 rpm until the *A*_600_ reached 0.5 to 0.6. At this point, the culture was induced with 1 mM isopropyl β-d-1-thiogalactopyranoside (IPTG) and further grown for 20 h at 24°C and 180 rpm. Cells were harvested by centrifugation at 5,000 × *g* for 10 min at 4°C. The cell pellet was resuspended in buffer A (50 mM Tris-HCl [pH 8.0], 20 mM imidazole, and 500 mM NaCl containing DNase I and lysozyme). The cells were lysed using an Emulsiflex C5 homogenizer (Avestin), and the lysate was cleared by centrifugation at 18,000 × *g* and 4°C for 45 min. The filtered supernatant was loaded onto a Ni affinity chromatography column pre-equilibrated with buffer B (50 mM Tris-HCl [pH 8.0], 20 mM imidazole, and 500 mM NaCl). Vag8 was eluted with a linear gradient of imidazole on a fast protein liquid chromatography (FPLC) system (ÄKTA Pure; GE Healthcare) using buffer B and buffer C (50 mM Tris-HCl [pH 8.0], 500 mM imidazole, and 500 mM NaCl). The eluted protein was dialyzed overnight into buffer D (50 mM Tris-HCl [pH 8.0] and 30 mM NaCl). The dialyzed protein was subjected to anion-exchange chromatography and eluted by a linear gradient of NaCl using buffer D and buffer E (50 mM Tris-HCl [pH 8.0] and 1 M NaCl). Purified Vag8 was concentrated, and the buffer was changed to buffer F (50 mM Tris-HCl [pH 8.0] and 150 mM NaCl) by ultrafiltration (Amicon Ultra; Merck-Millipore).

### Site-directed mutagenesis of Vag8.

Single mutations in Vag8 (H209A, Y234A, E237A, and W285A) were introduced using Q5 site-directed mutagenesis (New England Biolabs [NEB]). The Vag8 quadruple mutant (H209A Y234A E237A W285A) was produced by Gibson assembly of overlapping fragments containing the desired mutations using NEBuilder HiFi master mix (NEB). Purification of Vag8 mutants was done as described above for wild-type Vag8.

### Expression and purification of C1-INH.

A synthesized nucleotide fragment (codon optimized for Saccharomyces cerevisiae) encoding C1-INH amino acid residues 98 to 500 with Kozak and BiP signal sequence at the 5′ end (GeneArt; Thermo Scientific) was cloned using Gibson assembly (New England Biolabs) into pExpreS2-1 (ExpreS^2^ion Biotechnologies) plasmid, for protein production in *Drosophila* S2 cells, such that the mature recombinant protein had a His_6_ tag followed by a 3C protease cleavage site at the N terminus. The recombinant plasmid was transfected into S2 cells following the manufacturer’s protocol (ExpreS^2^ion Biotechnologies). Briefly, the recombinant plasmid was transfected into S2 cells, and a stable cell line was selected over a period of 4 weeks while the cells were cultured in Ex-Cell 420 medium (Sigma-Aldrich) supplemented with 10% (vol/vol) fetal bovine serum (FBS) and 4 mg/ml zeocin. The stable cell line was maintained in Ex-Cell 420 medium supplemented with 10% (vol/vol) FBS, penicillin-streptomycin, and amphotericin B and cultured at 25°C and 110 rpm. For protein purification, the stable cell line was split to a final cell density of 8 × 10^6^ cells/ml and cultured in Ex-Cell 420 medium, supplemented with penicillin-streptomycin and amphotericin B only, at 25°C and 110 rpm. The culture was centrifuged at 4,500 × *g* and 4°C for 30 min to collect the supernatant containing the recombinant protein 4 days after the split. The supernatant was filtered and incubated with His tag purification resin (Roche) overnight at 4°C with gentle mixing. The supernatant was then passed through a low-pressure gravity flow column to collect the resin, which was then washed with buffer F. The protein was eluted using buffer G (50 mM Tris-HCl [pH 8.0], 150 mM NaCl, and 500 mM imidazole) followed by dialysis into buffer D. The dialyzed protein was further purified using a MonoQ 10/30GL anion-exchange chromatography column (GE Healthcare) by a linear gradient of NaCl with buffer D and buffer E. Purified C-INH protein was concentrated, and the buffer was changed to buffer F (50 mM Tris-HCl [pH 8.0], 150 mM NaCl) by ultrafiltration (Amicon Ultra; Merck-Millipore).

### Preparation of Vag8–C1-INH complex.

The Vag8–C1-INH complex was prepared *in vitro* by incubating C1-INH in an ∼1.5 molar excess with Vag8 at room temperature for 10 min followed by purification using size exclusion chromatography on a S200pg 16/600 column (GE Healthcare). The eluted fractions were analyzed by SDS-PAGE followed by ultrafiltration to concentrate the protein complex.

### Size exclusion chromatography to assay the binding of Vag8 mutants to C1-INH.

A 100-μl mixture of C1-INH (20 mM) and Vag8 WT or mutant (10 mM) was prepared at room temperature and injected onto an S200 Increase 10/300GL column pre-equilibrated with 50 mM Tris-HCl–150 mM NaCl, pH 8.0. The samples were eluted at 0.4 ml/min, and 0.5-ml fractions were collected.

### Preparation of cryo-EM grids.

Four microliters of purified Vag8–C1-INH complex (0.5 mg/ml) was adsorbed to glow-discharged holey carbon-coated grids (Quantifoil 300 mesh; Au R1.2/1.3) for 10 s. Grids were then blotted for 3 s at 100% humidity at 8°C and frozen in liquid ethane using a Vitrobot Mark IV (FEI).

### Cryo-EM data collection, processing, and analysis.

Data were collected in counted superresolution mode on a Titan Krios G3 (FEI) operating at 300 kV with a BioQuantum imaging filter (Gatan) and K3 direct detection camera (Gatan) using either (i) a physical pixel size of 1.068 Å, a dose rate of 15 e−/pix/s, and an exposure of 4.23 s, corresponding to a total dose of 55.6 e^−^/Å^2^, or (ii) a physical pixel size of 0.832 Å, a dose rate of 13.9 e^−^/pix/s, and an exposure of 2.97 s, corresponding to a total dose of 59.6 e^−^/Å^2^. All movies were collected over 40 fractions.

Motion correction, dose weighting, contrast transfer function (CTF) estimation, particle picking, and extraction were performed in streaming mode during collection using SIMPLE 3.0 (42), as was 2D classification (42). *Ab initio* models were created in SIMPLE 3.0 using particles selected from chunks 1 and 2; further processing was performed in RELION 3.1 (43). The full workflow is described in Fig. 1; briefly, each data set underwent an initial round of 3D classification before 3D autorefine steps, beam tilt refinement, Bayesian polishing, and further rounds of 3D classification (43). Chunks of data were combined as described in Fig. 1 with the final volume calculated from 687,883 particles in C1. The resolution of the final volume is estimated as 3.6 based on an FSC criterion of 0.143 with the local resolution volume (calculated in RELION 3.1 [43]), demonstrating that much of the core of the complex is at a resolution of 3.5 or better.

### Data availability.

Coordinates and volumes have been deposited in the PDB and EMDB, respectively, with accession codes 7KAV and 11814.
